# Early Risk Stratification and Time-to-Cure Analysis in Patients with Residual Hemifacial Spasm After Microvascular Decompression

**DOI:** 10.3390/brainsci16090978

**Published:** 2026-09-16

**Authors:** Siyi Liu, Tao Du, Feng Hou, Zaiying Jiang, Liu He, Yunjian Huang, Bing Ni, Xueyuan Wang, Yunpeng Wang, Hongwei Zhu

**Affiliations:** 1 Xuanwu Hospital, Capital Medical University, Beijing 100053, China; lsymelody@126.com (S.L.); dtneuro1991@foxmail.com (T.D.); j15610471397@163.com (Z.J.); doctorheliu@163.com (L.H.); huangyunjian@mail.ccmu.edu.cn (Y.H.); nibing@xwh.ccmu.edu.cn (B.N.); 18600738633@163.com (X.W.); wangyunpeng53@hotmail.com (Y.W.); 2 Department of Neurosurgery, Beijing Neurosurgical Institute, Capital Medical University, Beijing 100071, China; houfeng123452022@163.com

**Keywords:** hemifacial spasm, microvascular decompression, delayed cure, persistent hemifacial spasm, nomogram, time to cure

## Abstract

**Highlights:**

**What are the main findings?**

- Among 140 patients with residual hemifacial spasm 24 h after microvascular decompression, 102 (72.9%) achieved delayed cure without reoperation. The median time to delayed cure was 30 days, and 95% of delayed cures occurred within 300 days after surgery.
- A prediction model incorporating ISIR, Cohen-post, and offending artery status showed good discrimination, with an area under the curve of 0.901.

**What are the implications of the main findings?**

- Persistent spasm shortly after surgery does not necessarily indicate surgical failure, as most patients eventually achieved symptom resolution without additional surgery.
- These findings may help clinicians counsel patients after surgery and guide follow-up decisions.

**Abstract:**

Background/Objectives: Microvascular decompression (MVD) is an effective treatment for hemifacial spasm (HFS), but some patients have residual spasms after surgery. Some later achieve delayed cure, whereas others have persistent HFS, making reoperation planning difficult. This study aimed to develop a prediction model for early risk stratification in patients with residual HFS after MVD. Methods: We retrospectively analyzed 500 consecutive patients with primary HFS who underwent MVD between November 2021 and August 2023. Among them, 140 patients with residual HFS at 24 h postoperatively were included. Clinical variables, intraoperative abnormal muscle response, Cohen grade at 24 h after surgery (Cohen-post), and Immediate Subjective Improvement Rate (ISIR) were collected. A multivariable logistic regression model was developed and presented as a nomogram, with bootstrap internal validation. Cox regression and Kaplan–Meier analyses were used to assess time to cure. Results: Mean follow-up was 21.9 months. Delayed cure occurred in 102 patients (72.9%), whereas 38 (27.1%) had persistent HFS. The final model included ISIR, Cohen-post, and offending artery status. The nomogram showed good discrimination (area under the curve 0.901). Patients with higher ISIR and lower Cohen-post recovered faster. Among patients with delayed cure, median time to cure was 30 days, and 95% achieved cure within 300 days after surgery. Conclusions: In patients with residual HFS at 24 h after MVD, a nomogram incorporating ISIR, Cohen-post, and offending artery status may help distinguish delayed cure from persistent HFS and may provide useful information on the likelihood of further recovery.

## 1. Introduction

Hemifacial spasm (HFS) is a movement disorder characterized by unilateral, involuntary, repetitive contractions of the facial muscles. Primary HFS is commonly caused by vascular compression at the root exit zone of the facial nerve [1]. Microvascular decompression (MVD) is currently the only curative treatment for HFS, in which the offending vessel is separated from the facial nerve root. Previous studies have reported an immediate cure rate after MVD of 71.8% (59.5–84%). In patients without immediate symptom resolution, many subsequently achieve delayed cure, whereas some continue to have persistent HFS.

Reoperation is an effective option for patients with unsatisfactory outcomes after the initial MVD, but the timing remains controversial. Early reoperation may be technically easier because adhesions and scarring around the Teflon padding are less severe, whereas delayed reoperation allows time for possible delayed cure [2,3,4]. In practice, the main difficulty is that, in the early postoperative period, it is often unclear which patients will gradually recover and which patients will have persistent symptoms. This uncertainty directly affects follow-up planning and decisions about whether reoperation should be considered [4,5].

Previous studies have described the natural course of delayed cure after MVD and identified factors associated with recovery [6,7,8]. However, for patients who still have HFS in the immediate postoperative period, whether the final outcome will be delayed cure or persistent HFS remains unclear, and whether this distinction can be made early is still uncertain. This question is highly relevant in clinical practice, because patients are usually fully awake by 24 h after surgery and often ask whether further recovery is still likely and, if so, how long it may take.

In this study, we focused specifically on patients with residual HFS at 24 h after MVD and aimed to develop an early prognostic model to distinguish delayed cure from persistent HFS using readily available early postoperative indicators, while also characterizing the time course of recovery in those who eventually achieved delayed cure.

## 2. Methods

### 2.1. Patients and Study Design

This was a single-center retrospective cohort study. Using data from patients with primary hemifacial spasm (HFS) who underwent microvascular decompression (MVD) at our institution, we developed a prediction model for long-term postoperative outcomes. This retrospective study was approved by the institutional ethics committee of Xuanwu Hospital, Capital Medical University (Approval No. KS20230222). The requirement for informed consent was waived because of the retrospective nature of the study.

### 2.2. Patient Selection

We retrospectively included consecutive patients with HFS who underwent first-time unilateral MVD at our center between November 2021 and August 2023. The study focused on patients with residual spasms in the early postoperative period; therefore, the inclusion criteria were: (1) primary unilateral HFS; (2) first-time MVD; and (3) residual HFS symptoms at 24 h postoperatively. The exclusion criteria were: (1) secondary HFS (e.g., cerebellopontine angle lesions causing compression of the facial nerve root exit zone); (2) severe postoperative complications (e.g., intracranial hemorrhage or other serious neurological adverse events); (3) reoperation during follow-up; and (4) loss to follow-up or incomplete follow-up data; no imputation was performed for missing outcomes. The last follow-up was completed in September 2024.

### 2.3. Collection of Data

Candidate predictors included demographic variables (age and sex), disease characteristics (symptom duration, affected side, medical history, and prior botulinum toxin treatment), comorbidities (e.g., hypertension, diabetes mellitus, and hyperlipidemia), intraoperative electrophysiological measures, and early postoperative clinical assessment indicators.

Intraoperative abnormal muscle response (AMR) was recorded by repeating provocation testing after decompression and placement of the padding material, before the end of surgery, and documenting the terminal status (whether AMR disappeared compared with the pre-decompression assessment: yes/no); this variable was included as a binary predictor. Early postoperative indicators included the Cohen grade at 24 h after MVD (Cohen-post) and the Immediate Subjective Improvement Rate (ISIR).

The Immediate Subjective Improvement Rate (ISIR) was defined in this study as an early postoperative subjective improvement indicator [9]: It was calculated from the patient’s self-reported percentage improvement within 24 h after surgery compared with the preoperative status (preoperative severity defined as 100%), based on follow-up or clinical course records. ISIR was entered as a continuous variable in the primary model. For descriptive survival analyses, ISIR was grouped using empirical thresholds (≤50%, 50–70%, and >70%).

HFS severity was assessed using the Cohen grading scale (0–4), and the assessment at 24 h postoperatively was defined as Cohen-post [5]. HFS severity was standardized on a 0–4 scale: grade 0, no spasm; grade 1, brief and localized spasm triggered by external stimuli; grade 2, mild spontaneous localized spasm without functional impairment; grade 3, moderate, more extensive and obvious spasm with mild functional impairment; and grade 4, severe, frequent and widespread spasm with marked functional impairment. In this study, “immediate postoperative” assessment was defined as the clinical evaluation at 24 h after MVD.

### 2.4. Outcome Definitions

The primary outcome was based on HFS status at the last follow-up.

- Delayed cure: residual symptoms at 24 h after surgery but complete resolution of HFS at the last follow-up.
- Persistent HFS: ongoing HFS at the last follow-up.

The logistic regression model was developed to distinguish delayed cure from persistent HFS at the last follow-up. Delayed cure was specified as the event (event = 1). Therefore, OR > 1 indicates a higher probability of delayed cure, whereas OR < 1 indicates a lower probability of delayed cure.

### 2.5. Development of Models and Survival Curves

Continuous variables are presented as mean ± standard deviation, and categorical variables as *n* (%). Between-group comparisons were performed using Welch’s *t*-test, the chi-square test, or Fisher’s exact test, as appropriate.

The primary prediction model was built using multivariable logistic regression. Odds ratios (ORs) with 95% confidence intervals (CIs) were reported. Model discrimination (C-index/ROC-AUC) and calibration were assessed. Internal validation was performed using bootstrap resampling (1000 iterations) for optimism correction and to assess model stability and calibration. Candidate predictors were considered based on clinical relevance, availability during the early postoperative period, statistical findings from univariable analysis, and model parsimony. The final model included ISIR, Cohen-post, and offending artery status. All analyses were performed in Python 3.9. A two-sided *p* value < 0.05 was considered statistically significant.

### 2.6. Time-to-Event Analysis (Time to Cure)

For the time-to-event analysis, survival time was defined as the interval from MVD to complete symptom resolution for patients with delayed cure, and as the interval from MVD to the follow-up time for patients with persistent HFS. Kaplan–Meier curves were used for visualization and to compare differences across key stratification variables (e.g., ISIR categories and Cohen-post strata). A Cox proportional hazards regression model was constructed after confirming the proportional hazards assumption, and between-group differences were assessed using the log-rank test.

### 2.7. Forest Plot Construction

Results of the multivariable logistic regression were presented as a forest plot, displaying ORs and their 95% confidence intervals on a logarithmic scale, with log(OR) = 0 as the null reference line. The forest plot was generated using GraphPad Prism version 10.1.2 (GraphPad Software, Boston, MA, USA).

## 3. Results

### 3.1. Baseline Characteristics

**Figure 1:**
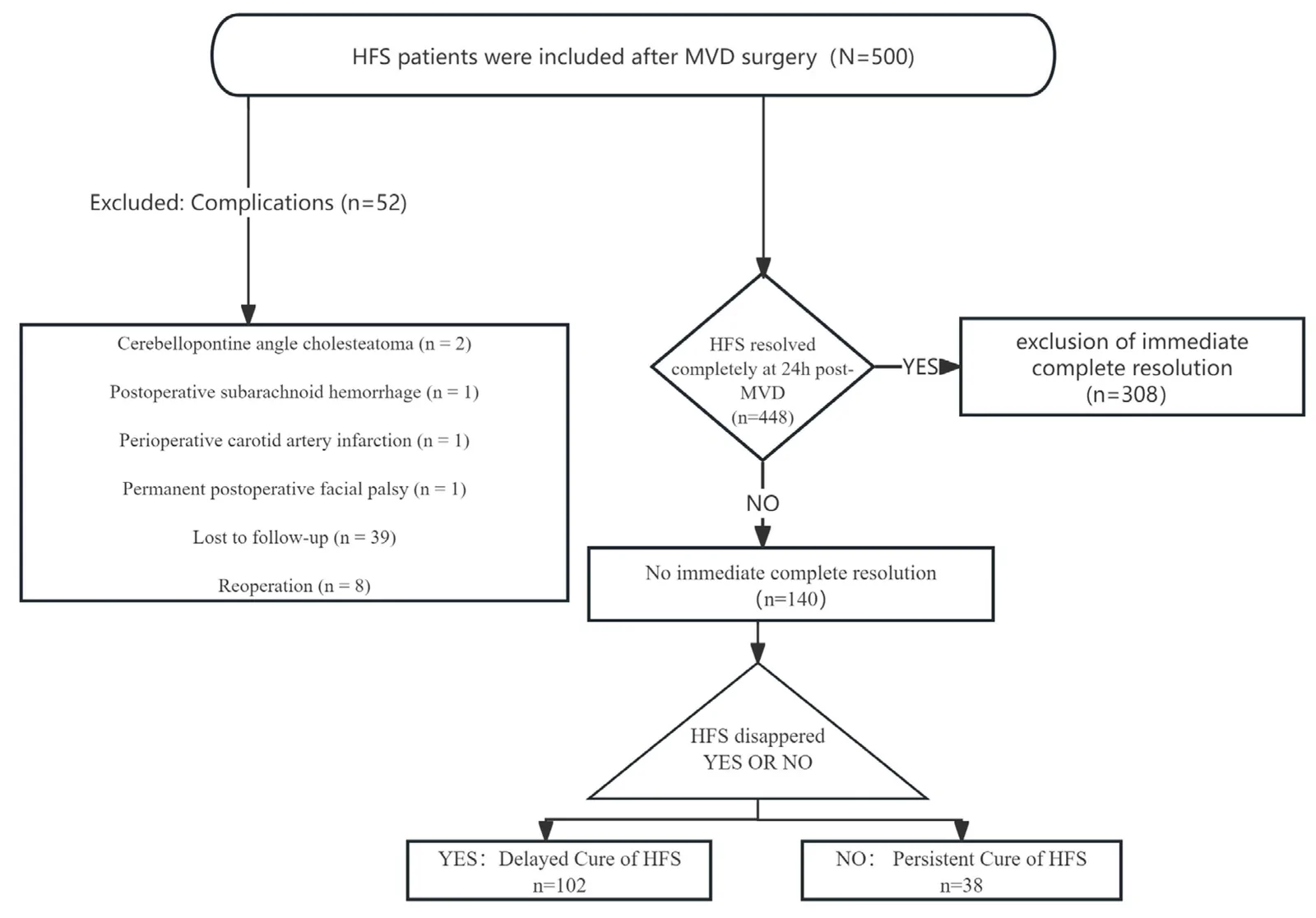
Study flowchart of patient selection and outcome classification after microvascular decompression (MVD). Patients with residual hemifacial spasm (HFS) at 24 h after MVD and complete follow-up were included and were classified as delayed cure or persistent HFS at the last follow-up.

Among 500 patients with primary HFS who underwent MVD, 360 were excluded according to the study criteria. These included two patients with cerebellopontine angle cholesteatoma, three with severe postoperative complications (postoperative subarachnoid hemorrhage, perioperative carotid infarction, or permanent postoperative facial palsy), 39 lost to follow-up, and eight who underwent repeat MVD during follow-up; the remaining 308 had complete symptom resolution on immediate postoperative assessment. Therefore, 140 patients with residual HFS on immediate postoperative assessment were included in the prognostic modeling cohort (Figure 1).

The mean (range) age of the 140 patients was 52.3 (28–75) years, and 93 (66.4%) were female. Right-sided spasm was present in 72 patients (51.4%). Most patients had a preoperative Cohen grade of 4 (*n* = 116, 82.9%), indicating severe spasm.

At 24 h after MVD, the mean Cohen grade decreased significantly from 3.81 to 1.97. The mean ISIR was 63.5% ± 24.6%. With a mean follow-up of 21.9 months (range, 12–36 months), 102 patients (72.9%) achieved delayed cure and 38 (27.1%) had persistent symptoms. Among patients with delayed cure, the median time to complete resolution was 30 days, and 95% achieved cure within 300 days after surgery.

All 140 eligible patients were divided into a delayed cure group and a persistent HFS group. ISIR (*t*-test) and Cohen-post (chi-square test) differed significantly between the two groups (*p* < 0.001), while no significant differences were observed in other baseline characteristics (Table 1).

### 3.2. Predictor Selection for the Model

Univariate logistic regression was performed to identify variables associated with delayed cure versus persistent HFS. Variables with *p* < 0.05 in univariate analyses included symptom duration, body mass index (BMI), age, ISIR, hyperlipidemia, affected side, preoperative Cohen grade (Cohen-pre), Cohen-post, number of offending arteries, and AMR.

**Figure 2:**
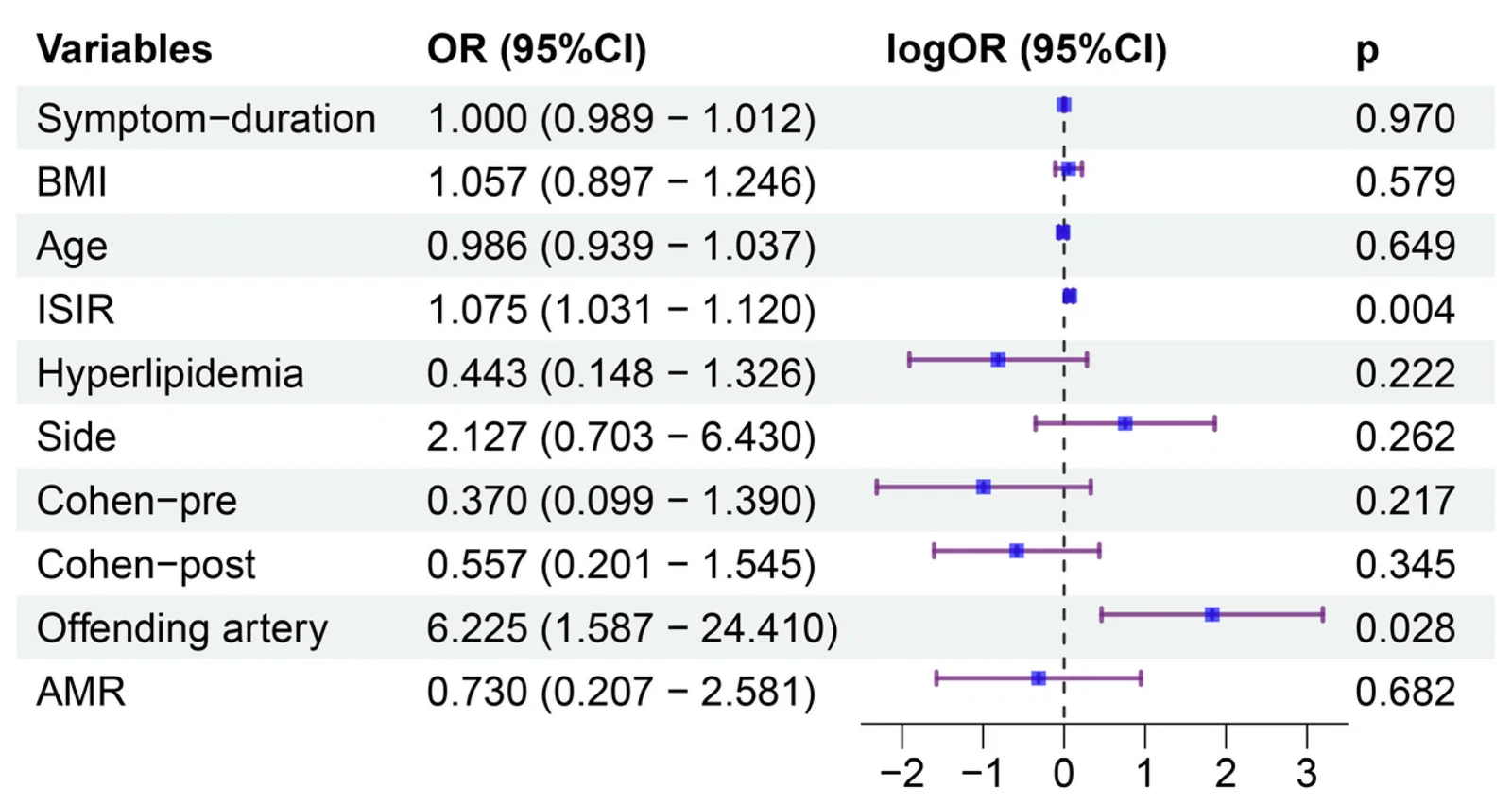
Forest plot of the multivariable logistic regression analysis: predictors of delayed cure of hemifacial spasm after MVD. The forest plot shows odds ratios (ORs) and 95% confidence intervals (CIs) for variables included in the multivariable logistic regression model. Blue squares indicate OR point estimates, and purple horizontal lines indicate 95% CIs. The vertical dashed line at log(OR) = 0 represents the null value. OR > 1 indicates a higher probability of delayed cure; OR < 1 indicates a lower probability of delayed cure.

In the multivariable logistic regression model (Figure 2), delayed cure was specified as the event (event = 1); thus, OR > 1 indicates a higher probability of delayed cure, whereas OR < 1 indicates a lower probability of delayed cure (i.e., relatively more likely persistent HFS). Considering clinical relevance, early postoperative availability, statistical findings from univariable analysis, and clinical interpretability, ISIR, Cohen-post, and single versus multiple offending arteries were included in the nomogram. ISIR and Cohen-post also differed significantly in baseline comparisons (Table 1). ISIR and Cohen-post were both retained in the model because they represented complementary patient-reported and clinician-rated assessments of early postoperative symptom improvement.

Detailed results of the univariable and multivariable logistic regression analyses are provided in Supplementary Table S1.

### 3.3. Nomogram Construction

**Figure 3:**
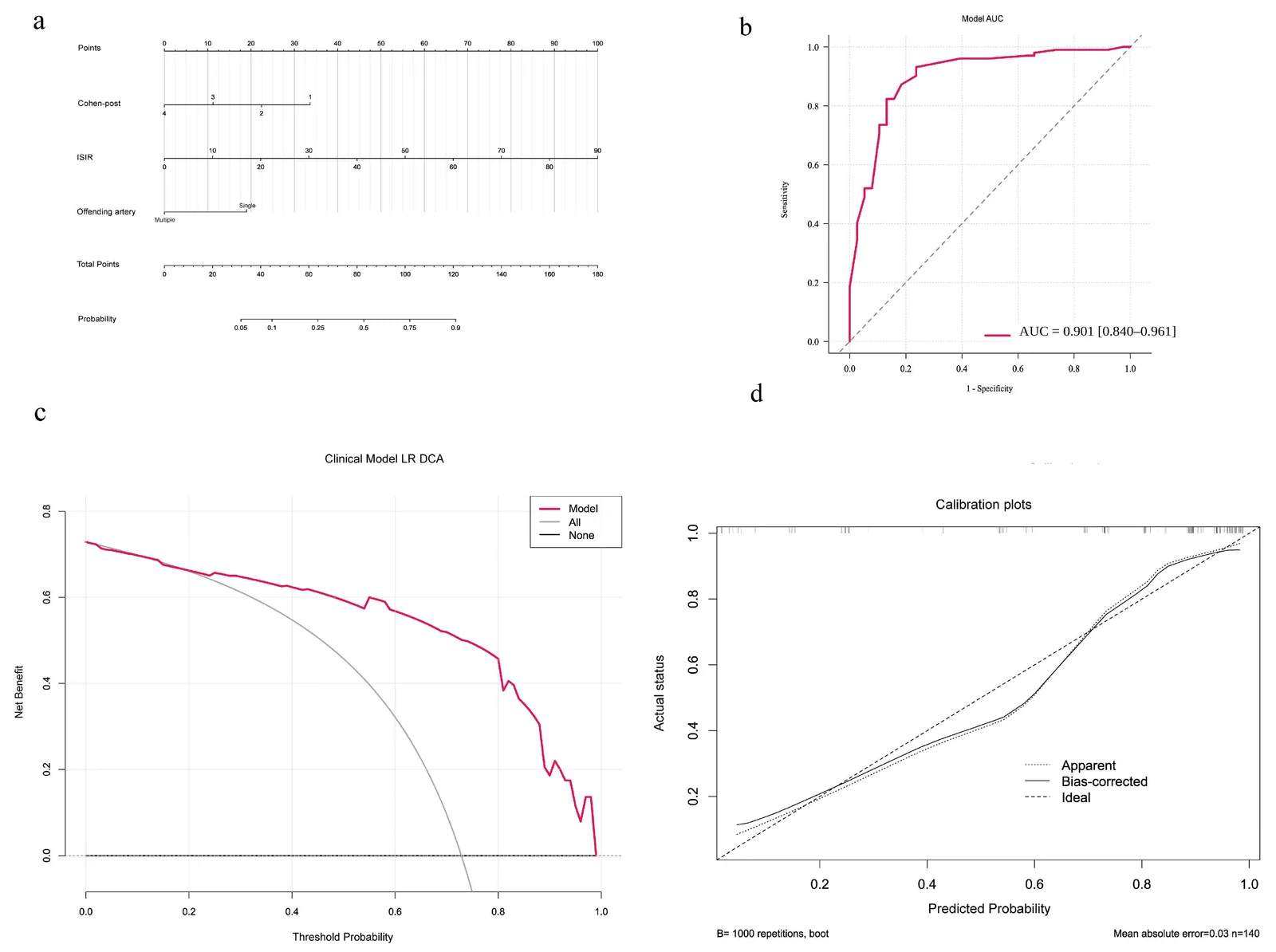
Nomogram based on logistic regression: (**a**) nomogram for predicting the probability of delayed cure among patients without immediate complete resolution after MVD; (**b**) receiver operating characteristic (ROC) curve and area under the curve (AUC) of the nomogram; (**c**) decision curve analysis (DCA) of the nomogram; (**d**) calibration curve of the nomogram.

Selected variables were entered into a logistic regression model to predict delayed cure versus persistent HFS in patients without immediate complete symptom resolution after MVD, and the model was presented as a nomogram (Figure 3a). The nomogram achieved an AUC of 0.901 (Figure 3b). Decision curve analysis (Figure 3c) and the calibration plot (Figure 3d) are shown. 

### 3.4. Survival Curves and Factor Analysis

**Figure 4:**
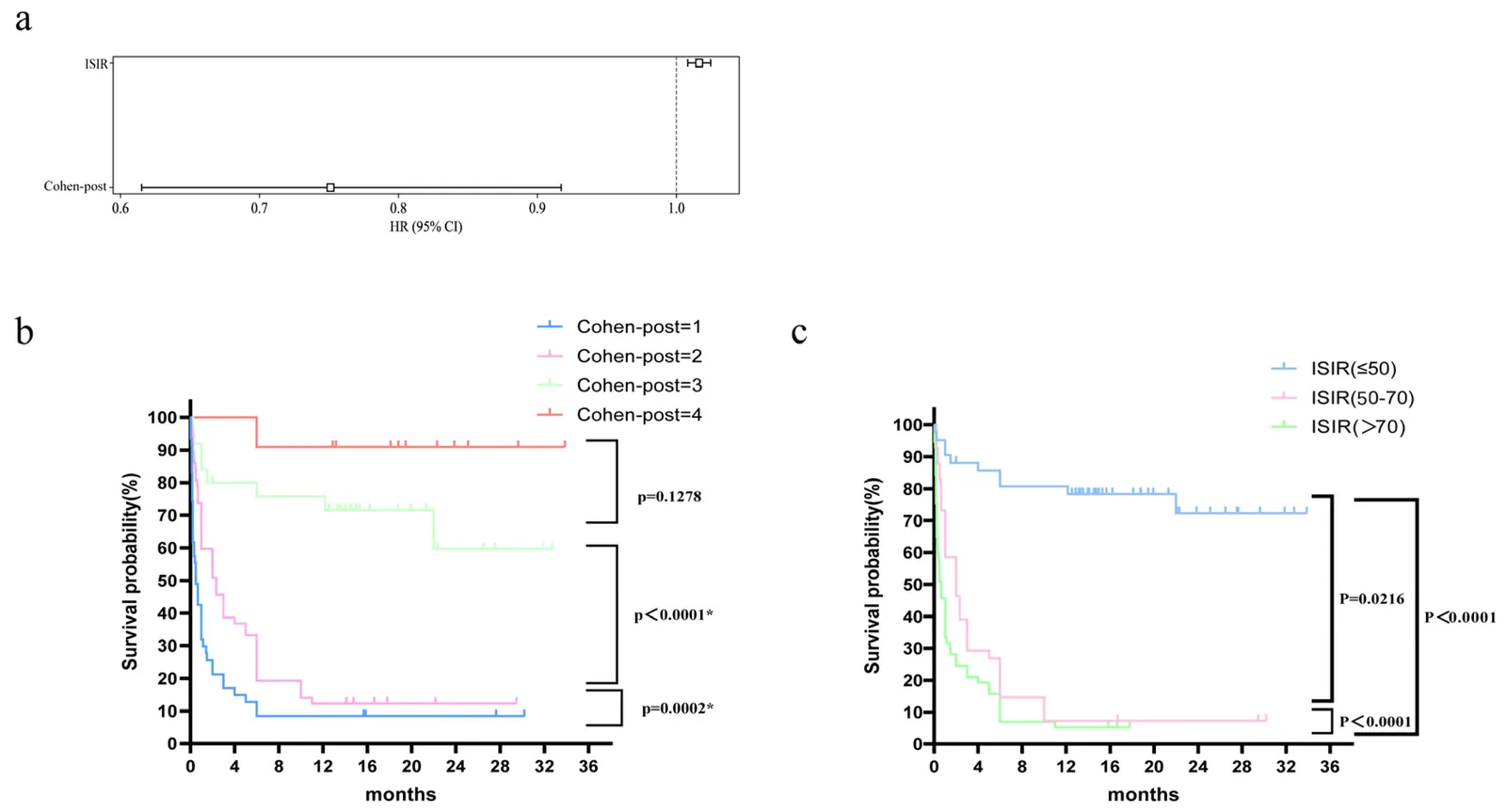
Kaplan–Meier survival curves for HFS patients without immediate complete resolution after MVD. (**a**) Forest plot of hazard ratios (HRs) from the multivariable Cox regression analysis; (**b**) Kaplan–Meier analysis stratified by Cohen-post grade (I, II, III, IV); (**c**) Kaplan–Meier analysis stratified by ISIR group (≤50%, 50–70%, >70%). The x-axis shows time (months), and the y-axis shows the persistent HFS rate. Both variables are independent predictors of delayed cure in the Cox model. * indicates a statistically significant pairwise log-rank comparison (*p* < 0.05).

Multivariable Cox regression analysis showed that ISIR and postoperative Cohen grade were statistically significant (*p* < 0.005) (Table 2; Figure 4a). Kaplan–Meier curves demonstrated significant differences in long-term outcomes across strata. Patients with higher ISIR recovered earlier than those with lower ISIR (log-rank *p* < 0.01), and patients with lower Cohen-post recovered earlier than those with higher Cohen-post (log-rank *p* < 0.05) (Figure 4b,c). In the Cox model, ISIR and Cohen-post remained independent predictors of delayed cure (Table 2).

## 4. Discussion

### 4.1. Summary of Key Findings

This study examined patients with residual HFS at 24 h after MVD, a subgroup in whom early postoperative management is challenging because delayed cure and persistent HFS cannot be reliably distinguished at that stage. Among 140 patients included in the prognostic analysis, 72.9% achieved delayed cure and 27.1% had persistent HFS at the last follow-up.

The final prediction model incorporated ISIR, Cohen-post, and offending artery status. ISIR and Cohen-post were practical symptom-based variables for early risk stratification in this cohort. Patients with ISIR > 70% and lower Cohen-post also had faster recovery in time-to-cure analyses.

### 4.2. Comparison with Previous Studies

Previous studies have shown that a substantial proportion of patients who do not achieve immediate relief after MVD ultimately experience delayed cure, and several clinical factors related to this process have been reported [2,3,6,8,10,11]. However, most of these studies are primarily descriptive and provide limited guidance for an important clinical question in patients with residual spasms: whether persistent spasm in the immediate postoperative period will eventually resolve or remain persistent, and whether this can be predicted early. A recent study developed a nomogram based on clinical multivariable factors to predict delayed cure after MVD, providing a reference for risk prediction in this field [6].

Nevertheless, most published work has focused on cure and delayed cure, with limited attention to patients who do not achieve delayed cure and ultimately have persistent spasms. Accordingly, the present study focused on patients with residual HFS on immediate postoperative assessment and performed early prognostic evaluation using routinely available perioperative variables. By integrating ISIR, postoperative Cohen grade, and offending artery status, our model helps distinguish delayed cure from persistent HFS. In addition, survival analyses of time to cure were used to show the course of delayed recovery, which may help answer a practical postoperative question: whether recovery is still likely and, if so, how long it may take.

### 4.3. Potential Mechanisms of Key Factors

The strong prognostic value of ISIR and Cohen-post may relate to the pathophysiology of HFS and the dynamic course of neural recovery after decompression. A higher ISIR accompanied by a lower Cohen-post may indicate adequate relief of neurovascular conflict, with rapid attenuation of ephaptic transmission and reduced excitability of the facial motor nucleus, thereby allowing gradual remyelination and synaptic remodeling; delayed cure may still occur even without immediate postoperative cure. This is consistent with proposed mechanisms of delayed cure, in which progressive reduction of facial nucleus excitability and gradual remyelination lead to symptom resolution despite the absence of immediate cure [1,4]. In contrast, a lower ISIR with a higher Cohen-post indicates limited early improvement and may reflect more prominent residual stimulation or more chronic nerve injury, with a relatively lower likelihood of delayed resolution. When multiple offending arteries are present, the anatomy is more complex and intraoperative handling is more challenging, and persistent symptoms or slower recovery may be more likely; studies on revision MVD have also suggested an association between anatomic complexity and the technical difficulty of reoperation [5,12]. It should be emphasized that predicted risk should be interpreted as a continuum: even with lower ISIR or higher Cohen-post, delayed cure may still occur in individual patients, and the symptom trends during follow-up should be considered in clinical decision-making. Recent work has also emphasized that offending vessel anatomy and the complexity of vascular decompression may influence postoperative outcomes, supporting the clinical relevance of anatomical factors in prognostic assessment [13].

### 4.4. Implications of Risk Stratification for Follow-Up and Reassessment

A central challenge in decisions about reoperation is that, early after surgery, it is often difficult to distinguish patients who will later achieve delayed cure from those who will have persistent symptoms. In this study, we combined ISIR, Cohen-post, and single versus multiple offending arteries into a nomogram-based risk estimate for early stratification. The purpose of this stratification is not to determine reoperation by itself, but to guide follow-up planning and reassessment timing. Patients with a higher predicted probability of delayed cure, especially those showing gradual symptom improvement, may be managed with observation and an adequate follow-up window. In contrast, patients with a higher predicted risk of persistent HFS—particularly those without clear improvement over time or with substantial symptom burden—may need closer follow-up and earlier reassessment (e.g., repeat clinical evaluation and further workup) to support decisions about further management.

Delayed symptom resolution should be recognized, but the timing of further intervention should not be based on a fixed waiting period alone. It should be based primarily on the trajectory of symptoms over time, rather than on follow-up timing alone, and should also take into account clinical findings and the patient’s quality of life and preferences.

### 4.5. Evidence and Clinical Considerations Regarding the Timing of Reoperation

Advocates of delayed reoperation favor conservative observation, arguing that this approach avoids misclassifying delayed cure as surgical failure and reduces unnecessary reoperations [6,10,11,14,15]. This viewpoint relies on observations of the natural history of delayed cure and often defines an adequate observation period using the median or maximum recovery time. In contrast, proponents of early reoperation emphasize the negative impact of persistent HFS on quality of life and patient anxiety [16,17]. From a technical perspective, earlier intervention is often easier to perform: operating before dense adhesions develop may allow clearer identification of neurovascular structures, facilitating more precise dissection and potentially reducing surgical risk [18]. In addition, studies have reported that repeat MVD performed by experienced surgeons has a safety profile comparable to that of the initial operation [12,18]. Our own experience also suggests that when the interval between the first and second operations is longer (e.g., more than 6 months), dense adhesions may form between the padding material and surrounding structures, increasing the difficulty of exposure and dissection [19].

**Figure 5:**
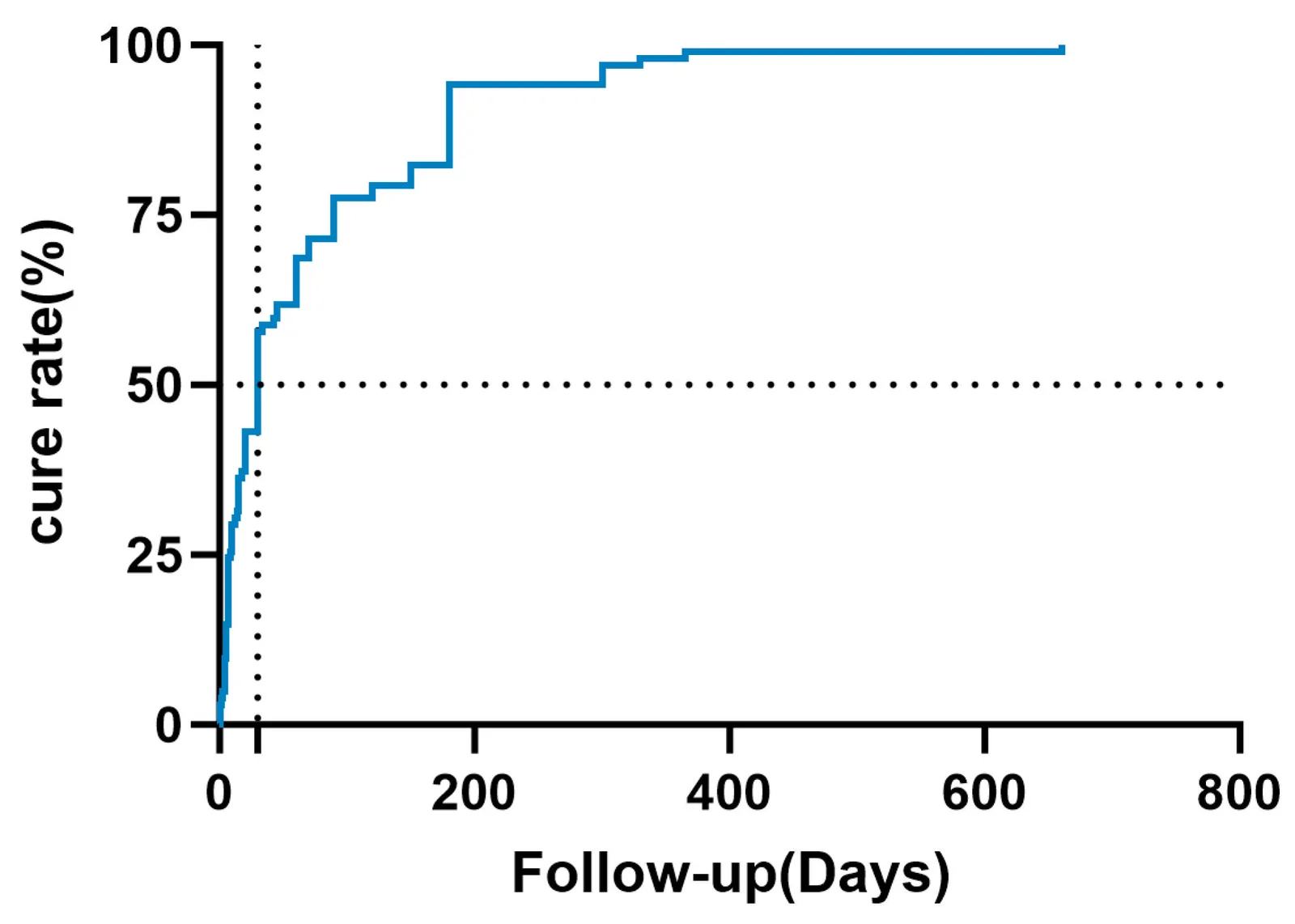
Reverse Kaplan–Meier curve showing the increase in the complete cure rate of HFS over time among patients with delayed cure (*n* = 102) (study population: patients without immediate complete resolution after MVD). The blue line shows the cumulative complete cure rate over time; the horizontal dotted line indicates 50% cure, and the vertical dotted line indicates the median time to cure (30 days).

The fundamental issue underlying this debate is that it is difficult to determine early after surgery which patients will ultimately achieve delayed cure and which will not. The key question is how to identify patients who require more active management at an early stage. In this regard, the risk stratification in the present study may help: for patients with a higher risk of persistent symptoms and substantial symptom burden, intensified follow-up and reassessment can be initiated earlier, and the feasibility of reoperation can be discussed after thorough risk–benefit communication; for patients with a higher probability of delayed cure, a longer observation window can be supported by the distribution of time to cure to avoid unnecessary reoperation. In this study, a reverse Kaplan–Meier curve was used to depict the distribution of time to cure among patients with delayed cure: approximately 95% achieved cure within 300 days after surgery, the median time to cure was 30 days, and the longest time to delayed cure was 366 days (Figure 5).

### 4.6. Predictive Factors Inform but Do Not Determine Reoperation Decisions

It should be emphasized that a predicted high risk of persistent HFS does not necessarily mean that reoperation is required. Repeat MVD is a major intervention with both physiological and psychological burden, and it is typically more technically demanding than the initial procedure. The model proposed in this study is intended to support, rather than replace, clinical judgment. When considering a second operation, surgeons should weigh the severity of residual spasms and their impact on quality of life, the patient’s tolerance and expectations, the acceptable level of surgical risk, and the surgeon’s experience with MVD and revision MVD. For some patients with persistent but tolerable symptoms, conservative management or continued observation may remain a reasonable option even when the predicted probability of delayed cure is low. In this sense, the model supports clinician–patient communication and shared decision-making, but it does not mandate early reoperation for all high-risk patients.

### 4.7. Limitations

This study has several limitations. First, this was a single-center retrospective study, and the findings mainly apply to a follow-up window of 12–36 months; the sample size was determined by case availability, and no prospective power calculation was performed. To mitigate overfitting related to the limited number of events, we used multivariable logistic regression as the primary model and performed internal validation with bootstrap resampling; therefore, the stability and generalizability of the model require further validation in larger samples and independent multicenter cohorts, and the time characteristics of ultra-delayed cure and recurrence should be further characterized.

Second, postoperative AMR monitoring was not routinely performed, particularly in patients with partial postoperative improvement who are often considered likely to experience delayed cure; however, the present model may help identify high-risk patients for earlier AMR reassessment [20,21]. Short-term postoperative MRA was also not routinely obtained, and even when imaging was performed, early postoperative studies were often difficult to interpret because the neurovascular relationship could be obscured by Teflon, hemostatic materials, and mild postoperative edema [22,23]. Intraoperative monitoring relied solely on AMR; prior reports have shown an approximately 20.86% discordance between intraoperative AMR disappearance and postoperative symptom relief, which may reflect the time required for facial nerve repair and recovery of nucleus excitability. Although intraoperative electrophysiological monitoring cannot guarantee complete decompression, it has been associated with better clinical outcomes [20,21]. In addition, we did not further subtype offending vessels into PICA/AICA/VA/BA, primarily for consistency in classification and standardization; future work with more rigorous standardized subtyping procedures will evaluate the incremental predictive value of specific vessel types. Finally, ISIR is an early postoperative observational indicator proposed in this study and offers simplicity and accessibility, but its measurement reliability and potential error require further evaluation and standardization in prospective studies. Psychological factors that may influence subjective symptom assessment were not routinely assessed or recorded in this retrospective cohort and should be considered in future prospective studies.

## 5. Conclusions

In patients with early postoperative residual hemifacial spasm after MVD, a nomogram based on ISIR, Cohen-post, and offending artery status may support risk stratification and follow-up reassessment, and may help early prognostic differentiation between delayed cure and persistent HFS.

## Figures and Tables

**Table 1:** Baseline characteristics stratified by final outcome (delayed cure vs. persistent HFS).

Variables	Feature	Overall (*n* = 140)	Delayed Cure (*n* = 102)	Persistent HFS (*n* = 38)	*p*
Age (years)	Mean ± SD	52.3 ± 10.3	52.0 ± 10.4	53.2 ± 10.1	0.545
BMI (kg/m^2^)	Mean ± SD	24.7 ± 3.5	24.8 ± 3.7	24.4 ± 3.0	0.527
Gender	M:F, *n* (%)	47:93 (33.6:66.4)	29:73 (28.4:71.6)	18:20 (47.4:52.6)	0.056
Symptom duration (months)	Mean ± SD	56.9 ± 56.6	59.0 ± 61.4	51.1 ± 41.0	0.379
Hypertension	Yes, *n* (%)	42 (30.0)	27 (26.5)	15 (39.5)	0.199
Diabetes	Yes, *n* (%)	14 (10.0)	11 (10.8)	3 (7.9)	0.758
Hyperlipidemia	Yes, *n* (%)	69 (49.3)	49 (48.0)	20 (52.6)	0.769
Botulinum toxin	Yes, *n* (%)	23 (16.4)	16 (15.7)	7 (18.4)	0.895
Tinnitus	Yes, *n* (%)	20 (14.3)	15 (14.7)	5 (13.2)	1.000
Side	L:R, *n* (%)	68:72 (48.6:51.4)	54:48 (52.9:47.1)	14:24 (36.8:63.2)	0.132
Cohen-pre grade	*n* (%)				0.191
	Grade 2	2 (1.4)	2 (2.0)	0 (0.0)	
	Grade 3	22 (15.7)	19 (18.6)	3 (7.9)	
	Grade 4	116 (82.9)	81 (79.4)	35 (92.1)	
Offending artery,	S:M, *n* (%)	117:23 (83.6:16.4)	85:17 (83.3:16.7)	32:6 (84.2:15.8)	1.000
AMR disappeared	Yes, *n* (%)	109(77.9)	80 (78.4)	29 (76.3)	0.969
Cohen-post grade	*n* (%)				<0.001
	Grade 1	48 (34.3)	44 (43.1)	4 (10.5)	
	Grade 2	57 (40.7)	49 (48.0)	8 (21.1)	
	Grade 3	24 (17.1)	8 (7.8)	16 (42.1)	
	Grade 4	11 (7.9)	1 (1.0)	10 (26.3)	
ISIR	Mean ± SD	63.5 ± 24.6	73.3 ± 16.6	37.1 ± 23.2	<0.001
Follow-up time (months)	Mean ± SD	21.9 ± 6.8 (12–36)	22.6 ± 6.9	20.2 ± 6.5	0.055

HFS: hemifacial spasm; BMI, body mass index; AMR, abnormal muscle response; ISIR, Immediate Subjective Improvement Rate. F, female; M, male; L, left; R, right; Cohen-pre, preoperative Cohen grade; Cohen-post, postoperative Cohen grade at 24 h after MVD, based on the Cohen scale for HFS severity; S, single offending artery; M, multiple offending arteries. Follow-up time was converted from days to months using days/30.

**Table 2:** Cox proportional hazards regression model for delayed cure in HFS patients without immediate complete resolution after MVD.

	Coefficient	HR	SE	Coefficient (95% CI)	HR (95% CI)	*z*	*p*
ISIR	0.02	1.02	0	0.01–0.02	1.01–1.02	3.92	<0.005
Cohen-post	−0.29	0.75	0.1	−0.49–−0.09	0.62–0.92	−2.81	<0.005

ISIR, Immediate Subjective Improvement Rate; Cohen-post, postoperative Cohen grade at 24 h after MVD, based on the Cohen scale for HFS severity.

## Data Availability

The individual-level clinical data generated and/or analyzed during the current study are not publicly available because they contain potentially identifiable patient information and are subject to ethical and institutional restrictions. Access to de-identified data may be considered by the corresponding author upon reasonable request and subject to approval by the relevant institutional authority.

## Supplementary Materials

The following supporting information can be downloaded at: https://www.mdpi.com/article/10.3390/brainsci16090978/s1, Table S1. Univariable and multivariable logistic regression analyses for delayed cure after microvascular decompression.

## Abbreviations

AICA, anterior inferior cerebellar artery; AMR, abnormal muscle response; AUC, area under the curve; BA, basilar artery; BMI, body mass index; CI, confidence interval; DCA, decision curve analysis; HFS, hemifacial spasm; HR, hazard ratio; ISIR, Immediate Subjective Improvement Rate; MRA, magnetic resonance angiography; MVD, microvascular decompression; OR, odds ratio; PICA, posterior inferior cerebellar artery; ROC, receiver operating characteristic; SD, standard deviation; VA, vertebral artery.
