# Supplementary material for: Early Risk Stratification and Time-to-Cure Analysis in Patients with Residual Hemifacial Spasm After Microvascular Decompression
Source: Brain Sci. 2026 Sep 16;16(9):978. doi: 10.3390/brainsci16090978 (PMC13604143; doi:10.3390/brainsci16090978)
Supplement: Supplementary file 1 [file brainsci-16-00978-s001.zip › brainsci-4513141-supplementary.pdf]

Supplementary Table S1 Univariable and multivariable logistic regression analyses for delayed cure after microvascular decompression

Variable	Univariable OR (95% CI)	P value	Multivariable OR (95% CI)	P value
Symptom duration	1.012 (1.006–1.018)	<0.001	1.000 (0.989–1.012)	0.970
BMI	1.042 (1.026–1.057)	<0.001	1.057 (0.897–1.246)	0.579
Age	1.019 (1.012–1.025)	<0.001	0.986 (0.939–1.037)	0.649
ISIR	1.024 (1.017–1.030)	<0.001	1.075 (1.031–1.120)	0.004
Gender	1.714 (0.986–2.980)	0.109	—	—
Hypertension	1.769 (1.000–3.130)	0.100	—	—
Diabetes	3.000 (1.002–8.980)	0.099	—	—
Hyperlipidemia	2.667 (1.621–4.389)	<0.001	0.443 (0.148–1.326)	0.222
Side	4.400 (2.472–7.830)	<0.001	2.127 (0.703–6.430)	0.262
Botulinum toxin	2.400 (1.000–5.760)	0.100	—	—
Cohen-pre	1.281 (1.170–1.402)	<0.001	0.370 (0.099–1.390)	0.217
Cohen-post	1.204 (1.043–1.391)	0.034	0.557 (0.201–1.545)	0.345
Offending artery	2.760 (1.879–4.051)	<0.001	6.225 (1.587–24.410)	0.028
AMR	2.857 (1.387–5.883)	0.017	0.730 (0.207–2.581)	0.682
Tinnitus	2.600 (1.094–5.178)	0.069	—	—

Abbreviations: OR, odds ratio; CI, confidence interval; ISIR, Immediate Subjective Improvement Rate; AMR, abnormal muscle response. $P < 0.05$ was considered statistically significant.

Note: Delayed cure was defined as the event (event = 1). Categorical variables were analyzed according to the reference categories used in the original model.
